# M. Demarquay on English Surgery

**Published:** 1863-09

**Authors:** 


### M. DEMARQUAY ON ENGLISH SURGERY.

M. Demarquay has recently published in the *Union Medicale* (No. 70) some of the impressions he derived concerning English Surgery during his visit to London as a juror of the Exhibition. Upon the whole, we do not complain of the estimate formed by our *confrere*, who sees that there is something at least to be learned from our hospitals and our practice; but one or two statements may be noticed. He seems to think that we administer chloroform in a far too free manner—an observation to be expected from a surgeon of the Paris hospitals, in which this assuager of suffering is often very imperfectly employed. The reason M. Demarquay assigns for such imperfect administration is of that idealistical kind we have become in the habit of regarding as peculiarly French. He says that when the English patient is brought completely under the influence of chloroform, "a painful feeling is produced when we behold placed upon the operating-table a human mass brought there solely to undergo the operations indicated by art. As chloroformization is continued during the entire period of the operation, and the patient is carried away while still under the influence of the anæsthetic, there results a complete isolation of the patient and the surgeon, which is truly painful. The man is effaced by such a mode of procedure, the operator becoming a mere instrument, and the patient mere matter for operation. Those moral and sympathetic ties, which become established between the operator and the operated prior to the operation, and at the moment when the patient returns to his senses, have no place in the English hospitals, where this mode of procedure prevails. In this point of view ours is the better practice." Aye, in this point of view; but what is it in the patient's point of view? Freedom from pain, rather than a "moral sympathetic tie," is, we take it, the grand point with him.

The construction and convenience of our operating theatres, their admirable illumination, the mode of transporting the patients to and fro, and the mode of dressing, receive great praise at the hands of M. Demarquay, contrasted as they are with what prevails in France. In comparing the results obtained in the two countries, he observed that we must bear in mind that the London patients can bear operations much better than the Parisians, who are of a far more nervous and impressionable temperament, whatever this may be due to, whether difference in race, in habits, or in manner of living. With respect to the diagnosis, he considers that French surgery is more minute, precise and affirmative, while in indications of treatment English surgery is more bold and more hasty. Thus, in respect to the numerous and remarkable examples of excision met with in the London hospitals, there has not first taken place in these cases that series of local and general treatment which would have been put into force in Paris. While English surgery is so proud of the results of these operations, French surgery is better pleased with avoiding such operations by medico-chirurgical treatment, and thus preserving their limbs in their entirety. In calculating the statistics, we may expect a greater proportion of favorable results from the English resections than from the French, as these latter are not resorted to until the last extremity, when all other means are exhausted. The true object of inquiry should be the diseases of the joints themselves; are they best treated by these more prompt and hasty operations, or by more varied and more expectant therapeutical agencies? But M. Demarquay finds that if, in some respects, London surgery is bolder than French, in others it is more timid. Thus, delicate operations for the removal of malignant tumors are much rarer in London than in Paris. We believe that this class of operations is of more common occurrence in our hospitals than M. Demarquay supposes, and that their greater rarity, compared with what prevails in Paris, is not due to any predominance of special hospitals, but to the increasing want of confidence entertained in their efficacy by our surgeons.—*Medical Times and Dublin Medical Press.*

